# Proximal Femoral Nail versus Dynamic Hip Screw Fixation for Trochanteric Fractures: A Meta-Analysis of Randomized Controlled Trials

**DOI:** 10.1155/2013/805805

**Published:** 2013-02-19

**Authors:** Xiao Huang, Frankie Leung, Zhou Xiang, Pei-Yong Tan, Jing Yang, Dai-Qing Wei, Xi Yu

**Affiliations:** ^1^Department of Orthopaedics, West China Hospital, Sichuan University, No. 37 Guoxue Xiang, Chengdu, Sichuan 610041, China; ^2^Department of Orthopaedics and Traumatology, Queen Mary Hospital, The University of Hong Kong, Hong Kong

## Abstract

*Background*. The purpose of this meta-analysis was to find out whether the proximal femoral nail was better than the dynamic hip screw in the treatment of trochanteric fractures with respect to operation time, blood transfusion, hospital stay, wound complications, number of reoperation, and mortality rate. *Methods*. All randomized controlled trials comparing proximal femoral nail and dynamic hip screw in the treatment of trochanteric fractures were included. Articles and conference data were extracted by two authors independently. Data was analyzed using RevMan 5.1 version. Eight trials involving 1348 fractures were retrieved. *Results*. Compared with DHS fixation, PFN fixation had similar operation time (95% CI: −15.28–2.40, *P* = 0.15). Blood loss and transfusion during perioperative time were also comparable between the two fixations (95% CI: −301.39–28.11, *P* = 0.10; 95% CI: −356.02–107.20, *P* = 0.29, resp.). Outcomes of hospital stay (95% CI: −0.62–1.01, *P* = 0.64), wound complication (95% CI: 0.66–1.67, *P* = 0.82), mortality (95% CI: 0.83–1.30, *P* = 0.72), and reoperation (95% CI: 0.61–1.54, *P* = 0.90) were all similar between the two groups. *Conclusion*. PFN fixation shows the same effectiveness as DHS fixation in the parameters measured.

## 1. Introduction

The incidence of the hip fracture has been rising with an aging population in many parts of the world, and the number of hip fractures is expected to reach 512,000 in the year 2040 [1]. 

Hip fractures include mainly trochanteric and femoral neck fractures, and the former was reported with a mortality rate ranging from 15% to 30% in America [2]. Surgical treatment with stable fixation allows early mobilization and reduces complications. There are two main types of fixations for trochanteric fractures, which are plate fixation and intramedullary implants [3, 4]. Dynamic hip screw (DHS) or sliding hip screw (SHS) has been the standard implant in treating trochanteric fractures [5–10]. However, when compared with the intramedullary implants, it has a biomechanical disadvantage because of a wider distance between the weight bearing axis and the implants [11]. The proximal femoral nail (PFN) introduced by the AO/ASIF group in 1998 has become prevalent in treating trochanteric fractures in recent years [12–15]. Although there were several reports showing benefits of proximal femoral nail [16–18], it was still associated with technical failures [19, 20]. The cost of PFN is also much more than DHS. 

Therefore, we conducted a meta-analysis to investigate whether there is a significant difference between PFN and DHS fixation in treating trochanteric fractures. The hypothesis is that PFN fixation is not more effective than DHS fixation in terms of decreasing operation time and blood transfusion, as well as reducing hospital stay, wound complication, reoperation, and mortality.

## 2. Methods

### 2.1. Search Strategies

The database for search included the Cochrane library, OVID, MEDLINE, EMBASE, and CNKI (China National Knowledge Infrastructure) from January 1998 to October 2012. Single or combinations of terms were searched as follows: dynamic hip screw, DHS, dynamic compression hip screw, sliding hip screw, SHS, proximal femoral nail, and PFN. 

### 2.2. Inclusion and Exclusion Criteria

Only prospective, randomized controlled trials (RCTs) and quasi-randomized controlled trials which compared the PFN fixation with the DHS fixation were selected. These studies enrolled patients with hip fractures classified as pertrochanteric or intertrochanteric without subtrochanteric extension by AO/OTA classification [21, 22]. Patients with pathological fractures, fractures associated with polytrauma, or patients with previous ipsilateral hip or femur surgeries were excluded. 

Targon PFN fixation was also included [25, 28], which offered a biaxial fixation of the proximal fragment [29], and was inserted in a similar method into the intramedullary cavity [10]. It was considered as a type of PFN implant.

If there were duplicates or multiple publications from the same study, which had overlap in original information, the most complete results should be chosen. If studies included had insufficient information, the authors were contacted for original data.

### 2.3. Data Extraction and Quality Assessment

Data were extracted by two experienced researchers independently. Different opinions were resolved by discussion. The assessment method which the Cochrane Handbook provided was used to assess the randomization, allocation concealment (according to whether allocation concealment was adequate, unclear, inadequate, or not used as a criterion to assess validity), blinding, and follow-up coverage of the studies included [30]. The three levels were assessed as follows: level A, all the criteria were adequate, which had a low risk of bias; level B, one or more of the criteria were not described, which had a moderate risk of bias; level C, one or more of the criteria were incorrect, inadequate, or not used, which had a high risk of bias.

### 2.4. Data Analysis

The weighted mean difference was calculated for continuous outcomes, and the relative risks (RRs) were calculated for dichotomous outcomes, with both adopted a 95% confidence interval (CI). The heterogeneity among studies was assessed using *I*-square (*I*²) test, Chi-square (*χ* ^2^) test, and Tau-square (*τ* ^2^) test. When there was no statistical heterogeneity (as judged by *χ* ^2^ test *P* ≥ 0.1 or *I*²<50%), a fixed effect model was adopted; otherwise, a random effect model was chosen. All analyses were performed by using the software Review Manager 5.1 [31]. A *P* value of less than 0.05 was considered statistically significant.

## 3. Results

**Figure 1:**
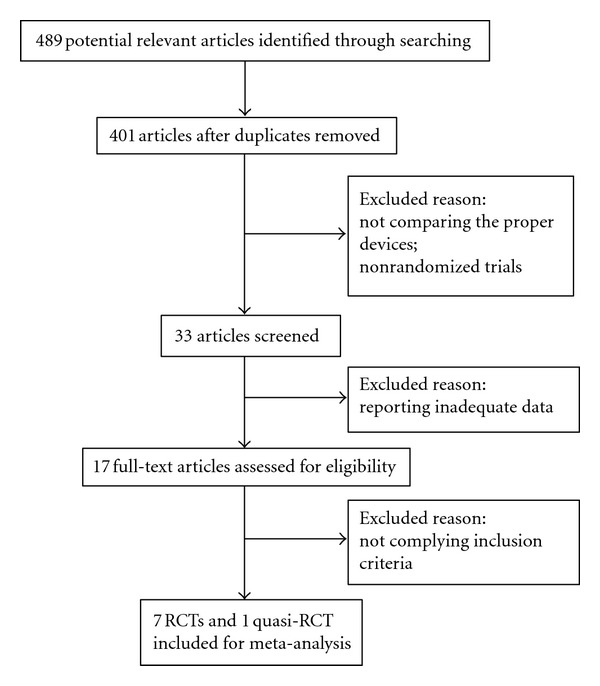
Flow diagram demonstrated methods for identification of studies and reasons for exclusion.

A total of 489 potentially relevant articles were retrieved: 25 from Cochrane library, 60 from MEDLINE, 114 from OVID, 137 from EMBASE, and 153 from CNKI. After excluding nonrandomized control trials and studies not comparing the proper implants and/or reporting inadequate data, eight randomized or quasi-randomized controlled trials were included (Figure 1). There were a total of 1348 patients who were predominantly elderly with trochanteric fractures. Five trials targeted Caucasian patients, and the other three targeted Asian ones. There were 675 patients treated by PFN and 673 by DHS. The baseline characterizations like age and gender were comparable in two groups (Table 1). Most studies evaluated operation time, blood loss, blood transfusion, wound complication, mortality, and reoperation. The quality of six studies included was level B for the allocation concealment, or the blinding was unclear according to the evaluation criteria mentioned above. The other two studies, one was level A which met all the criteria and another was level C because patients' medical record numbers, were used for allocation, and the allocation concealment was inadequate (Table 2).

### 3.1. Operation Time

**Figure 2:**
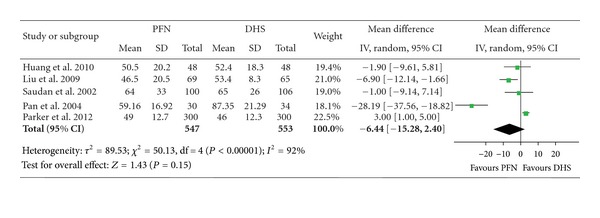
Comparison of operation time between PFN and DHS.

All eight studies provided data of operation time, but the data of five studies were eligible in the form of mean and standard deviation (SD). There were 1100 fractures included, 547 patients with the PFN fixation and 553 with the DHS fixation (Table 3). The heterogeneity test indicated a statistical evidence of heterogeneity (*χ*² = 50.13, *P* < 0.00001, *I*² = 92%). We pooled data by a random effect model which indicated that there was no statistical difference in operation time between the two groups. (mean difference: −6.44, 95% CI: −15.28–2.40, *P* = 0.15) (Figure 2).

### 3.2. Blood Loss and Transfusion

**Figure 3:**
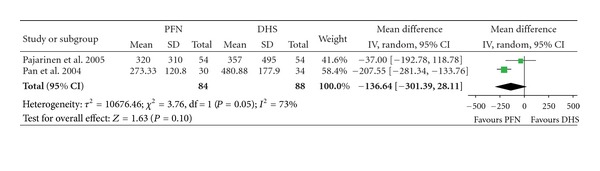
Comparison of blood loss between PFN and DHS.

**Figure 4:**
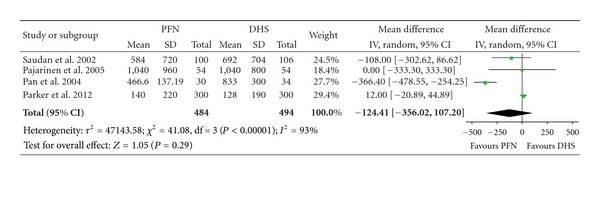
Comparison of blood transfusion between PFN and DHS.

There were two articles involving 172 fractures which provided data of blood loss (Table 3). The heterogeneity test indicated there was a statistical heterogeneity (*χ*² = 3.76, *P* = 0.05, *I*² = 73%), and the outcome shows no significant difference of blood loss with PFN than with DHS (Mean Difference: −136.64, 95% CI: −301.39–28.11, *P* = 0.10) (Figure 3). There were four articles included with 978 fractures providing data for blood transfusion. No significant difference in the amount of blood transfusion between the PFN group and the DHS group was found (Mean Difference: −124.41, 95% CI: −356.02–107.20, *P* = 0.29) (Figure 4).

### 3.3. Hospital Stay

**Figure 5:**
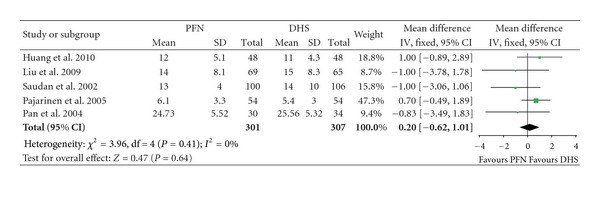
Comparison of hospital stay between PFN and DHS.

Five studies included data of hospital stay. There were a total of 608 patients, with 301 patients in the PFN group and 307 in the DHS group (Table 4). The heterogeneity test indicated no statistical heterogeneity (*χ*² = 3.96, *P* = 0.41, *I*² = 0%). Data pooled by a fixed effects model indicated that there was no statistical difference in hospital stay between the PFN group and DHS group (Mean Difference: 0.20, 95% CI: −0.62–1.01, *P* = 0.64) (Figure 5).

### 3.4. Wound Complication

**Figure 6:**
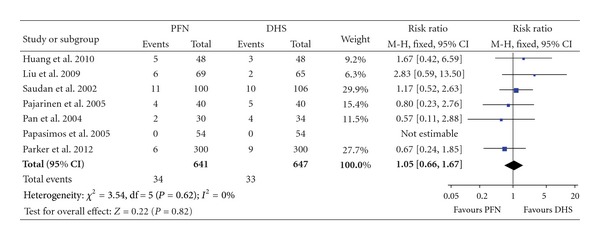
Comparison of wound complication between PFN and DHS.

Wound complications including wound infection, drainage, delayed healing, and hematoma were documented in seven studies while one showed no wound complication (Table 4). No statistical heterogeneity was presented by heterogeneity test (*χ*² = 3.54, *P* = 0.62, *I*² = 0%). Data pooled by a fixed effect model showed no statistical significant difference between the PFN group and the DHS group (RR: 1.05, 95% CI: 0.66–1.67, *P* = 0.82) (Figure 6).

### 3.5. Mortality

**Figure 7:**
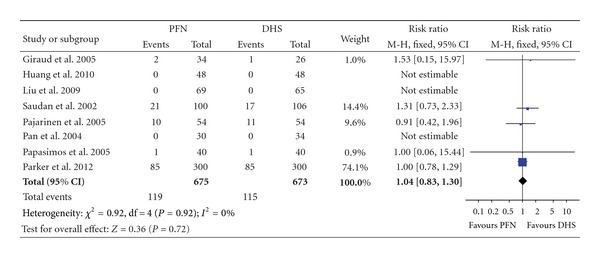
Comparison of mortality between PFN and DHS.

All eight studies provided data of mortality, with three of them observed no death in both groups during the period from operation to the last follow-up (Table 4). The average follow-up duration of these studies was 9.6 (3–28) months. The heterogeneity test indicated no statistical evidence of heterogeneity (*χ*² = 0.92, *P* = 0.92, *I*² = 0%), and data pooled by a fixed effect model indicated no statistical significant difference between the two groups (RR: 1.04, 95% CI: 0.83–1.30, *P* = 0.72) (Figure 7).

### 3.6. Reoperation

**Figure 8:**
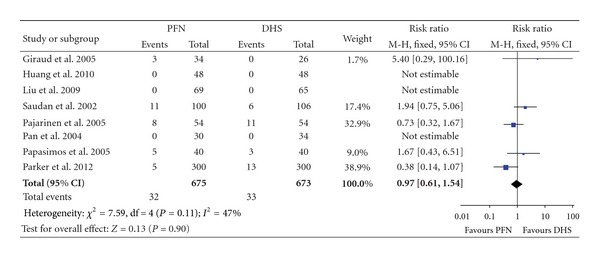
Comparison of reoperation between PFN and DHS.

The reasons for reoperation mainly were cut-out of femoral head, redisplacement of the fractures, breakage of the implant, and nonunion. The average follow-up duration of these studies was 9.6 (3–28) months. All eight articles provided data of reoperation, with three of them had no case of reoperation before the last follow-up (Table 4). The heterogeneity test indicated no statistical heterogeneity (*χ*² = 7.59, *P* = 0.11, *I*² = 47%), and data pooled by a fixed effect model indicated the outcome of reoperation was similar between the PFN group and the DHS group (RR: 0.97, 95% CI: 0.61–1.54, *P* = 0.90) (Figure 8).

## 4. Discussion

The optimal fixation device for trochanteric fractures is still controversial at present. Jones et al. [32] compared the intramedullary nail (IMN), which involved gamma nail, intramedullary hip screw (IMHS), and PFN, with sliding hip screw for treatment of extracapsular proximal femoral fractures. They concluded that there was no statistically significant difference in the cut-out rate between the IMN and SHS while total failure rate and reoperation rate were greater with IMN. Parker and Handoll [10] also compared gamma and other cephalocondylic intramedullary nails with extramedullary implants for extracapsular hip fractures in adults. In their systematic review the authors enrolled four studies which included PFN and Targon PF nail compared with SHS. The authors concluded that there was no significant difference between the groups in outcomes of blood loss and transfusion, fixation complications, and post-operation complications and hospital stay.

This meta-analysis included eight RCTs, some of which were recently published and not included in previous meta-analysis and systematic review. We were able to show that PFN fixation and DHS fixation had similar effectiveness in the treatment of trochanteric fractures.

The analysis of operation time showed no significant difference between the two groups. But there was a notable heterogeneity, which could probably be explained by the different levels of experience of surgeons, and the duration of PFN fixation could be shortened as surgical skills improved.

This paper showed no significant difference of blood loss and blood transfusion between the two implants. We could only find detailed outcomes related to blood loss and blood transfusion of four randomized studies from which data could be extracted. We enrolled studies of Pan et al. [24] and Pajarinen et al. [18] for analyzing blood loss and studies of Pan et al. [24], Pajarinen et al. [18], Parker et al. [28], and Saudan et al. [23] for blood transfusion. A sensitivity test was performed, which showed that, in blood transfusion, the two groups were still similar. In practice, various counting methods of blood loss were used in different hospitals, and surgeons usually estimate the blood loss. That could explain the statistic significant difference of heterogeneity (*P* = 0.01, *I*² = 77%), which made it hard to draw any conclusion.

The intention-to-treat analysis (ITT) was used in the analysis of mortality and reoperation to reduce the withdrawal bias [33–36]. Furthermore, we tried to contact the authors of these studies for additional information but only received one response for checking the data of their research. This paper listed three cases of hematoma, one case of superficial wound infection, and one case of delayed wood healing in wound complications, but the author added it up to four [26]. Nevertheless, the outcome was still similar between the two groups when we performed a sensitivity test. It would be desirable if all the data from the studies were fully reported in a standard format, so that a larger sample can be analyzed to reduce bias. 

There were some limitations in this meta-analysis. The number of studies included was not so adequate which just had eight studies involving 1348 fractures, and the quality of the trials was generally low. We intended to perform a subgroup analysis based on AO/OTA classification of the trochanteric fracture initially. Unfortunately, not all the studies provided precise classification of patients and had inadequate outcome data for extraction. Furthermore, different follow-up duration of included studies also reduced the power of our research.

In summary, PFN and DHS are equally effective in the treatment of trochanteric fractures. With future modifications to these two types of implants, more high-quality randomized controlled trials and further studies are needed to investigate whether these changes can lead to different outcomes. 

## Figures and Tables

**Table 1:** Characteristics of the included studies.

Studies	Ages (years)		Men (%)	Setting	Follow-up (months)	Fracture type (OTA, 31-)	Year
	PFN	DHS					
Saudan et al. [23]	83 ± 9.7	83.7 ± 10.1	22.3	Switzerland	12	A1, A2	2002
Pan et al. [24]	70 ± 6.8	69 ± 7.1	75	China	16 (12–28)	A1, A2, A3	2004
Pajarinen et al. [18]	80.9 ± 9.1	80.3 ± 10.8	25	Finland	4	A(1.1-1.2, 2.1-2.2, others)	2005
Giraud et al. [25]	81 ± 12.8	82 ± 9.8	23	France	3	A1, A2, A3	2005
Papasimos et al. [26]	79.4	81.4	38.8	Greece	12	A2, A3	2005
Liu et al. [27]	76 ± 4.3	78 ± 4.5	29.1	China	9	A1, A2	2009
Huang et al. [17]	75 ± 5	77 ± 5	26	China	9	A1, A2	2010
Parker et al. [28]	81.4 (27–104)	82.4 (26–104)	20.2	UK	12	A1, A2, A3, B2.1	2012

PFN: proximal femoral nail; DHS: dynamic hip screw; OTA: Orthopaedic Trauma Association.

**Table 2:** Methodological quality of included studies.

Studies	No. of fractures		Randomization	Allocation concealment	Blinding	Loss to follow-up	Level
	PFN	DHS					
Saudan et al. [23]	100	106	Adequate	Not described	Not described	Yes	B
Pan et al. [24]	30	34	Inadequate	Inadequate	Adequate	Yes	C
Pajarinen et al. [18]	54	54	Adequate	Adequate	Not described	Yes	B
Giraud et al. [25]	34	26	Adequate	Not described	Not described	Yes	B
Papasimos et al. [26]	40	40	Not described	Not described	Not described	Yes	B
Liu et al. [27]	69	65	Not described	Not described	Not described	Yes	B
Huang et al. [17]	48	48	Adequate	Adequate	Not described	Yes	B
Parker et al. [28]	300	300	Adequate	Adequate	Adequate	Yes	A

Loss to follow-up: reported patients loss to follow-up.

**Table 3:** Intraoperative outcomes of the two groups.

Studies	Operation time (min)		Blood loss (mL)		Blood transfusion (mL)^a^	
	PFN	DHS	PFN	DHS	PFN	DHS
Saudan et al. [23]	64 ± 33	65 ± 26	NA	NA	584 ± 720	692 ± 704
Pan et al. [24]	59.16 ± 16.92	87.35 ± 21.29	273.33 ± 120.8	480.88 ± 177.90	466.6 ± 137.19	833 ± 300
Pajarinen et al. [18]	55 (35–200)	45 (20–105)	320 ± 310	357 ± 495	1040 ± 960	1040 ± 800
Giraud et al. [25]	35	42	410	325	NA	NA
Papasimos et al. [26]	71.2 (60–240)	59.2 (40–100)	265	282.4	NA	NA
Liu et al. [27]	46.5 ± 20.5	53.4 ± 8.3	136	152	NA	NA
Huang et al. [17]	50.5 ± 20.2	52.4 ± 18.3	202.5	225	200	200
Parker et al. [28]	49 ± 12.7	46 ± 12.3	NA	NA	140 ± 220	128 ± 190

^a^Blood transfusion (mL) had a unit conversion from original articles; NA: not available.

**Table 4:** Postoperative outcomes of the two groups.

Studies	Hospital stay (days)		Wound complication^b^		Mortality^b^		Reoperation^b^	
	PFN	DHS	PFN	DHS	PFN	DHS	PFN	DHS
Saudan et al. [23]	13 ± 4	14 ± 10	11 (11%)	10 (9.43%)	21 (21%)	17 (16.04%)	11 (11%)	6 (5.66%)
Pan et al. [24]	24.73 ± 5.52	25.56 ± 5.32	2 (6.67%)	4 (11.76%)	0 (0%)	0 (0%)	0 (0%)	0 (0%)
Pajarinen et al. [18]	6.1 ± 3.3	5.4 ± 3.0	0 (0%)	0 (0%)	10 (18.52%)	11 (20.37%)	8 (14.81%)	11 (20.37%)
Giraud et al. [25]	11	11	NA	NA	2 (5.89%)	1 (3.85%)	3 (8.82%)	0 (0%)
Papasimos et al. [26]	8.8	9.9	4 (10%)	5 (12.5%)	1 (2.5%)	1 (2.5%)	5 (12.5%)	3 (7.5%)
Liu et al. [27]	14 ± 8.1	15 ± 8.3	6 (8.70%)	2 (3.08%)	0 (0%)	0 (0%)	0 (0%)	0 (0%)
Huang et al. [17]	12 ± 5.1	11 ± 4.3	5 (10.42%)	3 (6.25%)	0 (0%)	0 (0%)	0 (0%)	0 (0%)
Parker et al. [28]	21.2 (1–408)	18.7 (1–141)	6 (2%)	9 (3%)	85 (28.33%)	85 (28.33%)	5 (1.67%)	13 (4.33%)

^b^The intention-to-treat analysis (ITT) was used in the analysis to reduce the withdrawal bias.

NA: not available.
